# Solitary fibrous tumor – clinicopathologic, immunohistochemical and molecular analysis of 28 cases

**DOI:** 10.1186/s13000-014-0224-6

**Published:** 2014-11-29

**Authors:** Rob JC Vogels, Myrella Vlenterie, Yvonne MH Versleijen-Jonkers, Emiel Ruijter, Elise M Bekers, Marian AJ Verdijk, Monique M Link, Johannes J Bonenkamp, Winette TA van der Graaf, Pieter J Slootweg, Albert JH Suurmeijer, Patricia JTA Groenen, Uta Flucke

**Affiliations:** Department of Pathology, Radboud University Medical Center, P.O. Box 9101, 6500 HB Nijmegen, The Netherlands; Department of Medical Oncology, Radboud University Medical Center, Nijmegen, The Netherlands; Department of Pathology, Rijnstate Hospital, Arnhem, The Netherlands; Department of Surgical Oncology, Radboud University Medical Center, Nijmegen, The Netherlands; Department of Pathology, University Medical Center Groningen, University of Groningen, Groningen, The Netherlands

**Keywords:** Solitary fibrous tumor, Hemangiopericytoma, *NAB2-STAT6* fusion, RT-PCR, STAT6 immunohistochemistry, EGR1 immunohistochemistry, Soft tissue

## Abstract

**Background:**

Solitary fibrous tumor is a mesenchymal tumor of fibroblastic type, which can affect any region of the body. Recently, a recurrent gene fusion *NAB2-STAT6* has been identified as molecular hallmark. The *NAB2-STAT6* fusion leads to EGR1 activation and transcriptional deregulation of EGR1-dependent target genes and is a driving event in initiation of SFT. In this study, we report the clinicopathologic and RT-PCR findings and evaluated expression of STAT6 and EGR1 protein in a cohort of 28 SFTs.

**Methods:**

28 patients with a median age of 54 years were included with SFTs originating at different sites, most occurring in the lung and pleura (9, 32%), 5 in soft tissues of the lower extremities (18%) and 5 in the head and neck (18%). For detection of the *NAB2-STAT6* fusion gene, RT-PCR was performed using RNA extracted from formalin-fixed and paraffin-embedded tissues. Immunohistochemistry was performed on all cases with antibodies against STAT6 and EGR1.

**Results:**

All patients were treated by surgery, 3 with adjuvant chemo- or radiotherapy. Follow-up data of 18 patients could be obtained of which 2 patients died of metastatic disease 13 months and 52 years after first diagnosis. Sixteen patients have no evidence of disease with a median follow up of 29.5 months (range 7 – 120 months). *NAB2-STAT6* fusion transcripts were found in 19/28 cases (68%). The most common fusion was between *NAB2* exon 4 and *STAT6* exon 3 (11/19, 58%), mainly occurring in pleuropulmonary lesions. All cases showed strong nuclear expression of STAT6 (28/28, 100%) while EGR1 showed low-level variable nuclear expression in all samples, comparable with the EGR1 expression results of the control group.

**Conclusions:**

The identification of the *NAB2-STAT6* fusion in SFTs can provide important diagnostic information, especially in cases with aberrant morphology or when biopsy material is limited. STAT6 immunohistochemistry is another useful tool in diagnosing SFT. EGR1 immunohistochemistry indicates low-level protein expression in accordance with EGR1 activation due to distorted NAB2 activity.

**Virtual slides:**

The virtual slide(s) for this article can be found here: http://www.diagnosticpathology.diagnomx.eu/vs/13000_2014_224

## Background

Solitary fibrous tumor (SFT) is a mesenchymal tumor of fibroblastic type that can affect virtually any region of the body [1,2]. The neoplastic cells are arranged in a patternless architecture with alternating hypo- and hypercellular areas and a prominent branching vasculature. These lesions occur predominantly in middle-aged adults with equal gender distribution [1]. Most tumors present as well defined, slow growing masses, which can be cured by surgery. A small percentage of SFTs, between 10-20%, behave in a more aggressive way, with local recurrence and/or distant metastasis for which systemic therapy (chemotherapy or targeted treatment with e.g. sunitinib) can be given [1,3-5]. Prediction of behavior is difficult, with tumor size above 15 cm, positive surgical margins, tumor site and high mitotic count (>4/10 high power fields, HPF) being the most useful indicators for malignancy [3,6-8].

Recently, a recurrent gene fusion *NAB2-STAT6* has been identified as molecular hallmark of SFT, encoding a chimeric protein that combines the EGR-binding domain of NAB2, a repressor of early growth response (EGR) transcription factors that regulate differentiation and proliferation, with the transactivation domain of STAT6, a transcription factor that mediates cytokine signaling [2,9]. Molecular detection of the fusion gene and immunohistochemical expression of nuclear STAT6 can be helpful in diagnosing SFT, especially in cases not unequivocally classifiable [2,10-13].

In this study, molecular analysis and immunohistochemical staining of STAT6 protein was performed in 28 cases of SFT. In addition, as the *NAB2-STAT6* fusion leads to EGR1 (early growth response protein 1) activation and transcriptional deregulation of EGR1-dependent target genes, we immunohistochemically evaluated the expression of EGR1 in our tumor samples in order to semi-quantify EGR1 protein levels in SFT [2,14].

## Methods

### Tissue samples and immunohistochemistry

Twenty-eight cases were selected from the (referral) files of the authors between 01–2002 and 08–2014 and slides were reviewed by two of them (UF, PS). The study was performed in accordance with the Code of Conduct of the Federation of Medical Scientific Societies in the Netherlands. The tissue was fixed in 4% buffered formalin, routinely processed and embedded in paraffin; 4 μm thick sections were stained with hematoxylin and eosin and immunohistochemistry was performed using commercially available antibodies listed in Table 1.

**Table 1:** **Details of used antibodies**

**Antibody**	**Clone**	**Dilution**	**Source**
STAT6	YE361	1:80	ABCam, Cambridge, UK
EGR1	T.160.5	1:50	ThermoFisher Scientific, Waltham, USA
CD34	QBEnd/10	1:80	Immunologic, Duiven, the Netherlands
CD99	O13	1:150	ThermoFisher Scientific, Waltham, USA
EMA	E29	1:250	DAKO, Glostrup, Denmark
SMA	1A4	1:30000	Sigma, Saint Louis, USA
S-100	polyclonal	1:10000	DAKO, Glostrup, Denmark
Bcl-2	124	1:80	DAKO, Glostrup, Denmark

Antigen retrieval was performed using EDTA buffer, pH 9,0 for 10 minutes at 95°C and 10 minutes blocking with 3% H2O2 in methanol. The primary antibodies (dilutions presented in Table 1) were added for 1 hour at room temperature. Secondary antibody Poly-HRP Gam/R/Ra; Immunologic was applied for 30 minutes at room temperature. The chromogenic substrate Bright DAB; Immunologic was applied for 7 minutes at room temperature.

Cases were scored positive for STAT6, CD34, CD99, EMA, SMA, Bcl-2 and S100 when at least 50% of tumor cells showed strong staining. EGR1 was scored using a six-point scale (0 = negative; 1 = <5% nuclei +; 2 = 5-25% nuclei +; 3 = 26-50% nuclei +; 4 = 51-75% nuclei +; 5 = 76-100% nuclei +). As (negative) control samples for STAT6 and EGR1, some possible mimickers of SFT were stained (5 dedifferentiated liposarcomas, 5 deep benign fibrous histiocytomas, 5 sarcomatoid mesotheliomas, 7 low grade fibromyxoid sarcomas, 5 schwannomas, 4 malignant peripheral nerve sheath tumors, 5 gasto-intestinal stroma cell tumors, 5 synovial sarcomas and 6 leiomyomas). Endothelial nuclei served as an internal positive control for EGR1. Clinical information, where available, was obtained from the hospital records.

### Reverse transcriptase-Polymerase chain reaction (RT-PCR)

RNA was extracted from formalin-fixed and paraffin-embedded tissues (FFPE) using RNA-Bee-RNA isolation reagent (Bio-Connect BV, Huissen, the Netherlands) according to standard procedures. RNA quantity and quality were determined by a NanoDrop measurement (Fisher Scientific, Landsmeer, the Netherlands) and subsequently, cDNA synthesis was performed using Superscript II (Invitrogen Life Technologies Europe, Bleiswijk, the Netherlands) and random hexamers (Promega Nederland, Leiden, the Netherlands).

The cDNA was tested by the reverse transcription-polymerase chain reaction (RT-PCR) for the HMBS (hydroxymethylbilase synthase) housekeeping gene using the primers forw150 5′-TGCCAGAGAAGAGTGTGGTG-3′, rev150 5′-ATGATGGCACTGAACTCCTG-3′, forw250 5′- CTGGTAACGGCAATGCGGCT-3′, rev250 5′- TTCTTCTCCAGGGCATGTTC-3′.

For the detection of the NAB2-STAT6, three primers in NAB2 (NM_005967.3) exon3 forw 5′- CAAGTAGCCCGAGAGAGCAC-3′, exon4 forw 5′- CTCCACTGAAGAAGCTGAAAC-3′ and exon6 forw 5′-CTGTGTGCCTGCGAAGCC-3′ were used in combination with three STAT6 (NM_001178078.1) primers: rev 5′-GGGAAAGTCGACATAGAGCC-3′ (exon3), rev 5′-GAGCTGAGCAAGATCCCGG-3′ (exon17) and rev 5′-TTCCACGGTCATCTTGATGG-3′ (exon18). The PCR products were analyzed by agarose gel electrophoresis. The sequence of the differently sized PCR-products was obtained by Sanger sequencing and confirmed presence of the fusion gene.

## Results

### Clinical data

Clinical data are summarized in Table 2. Twenty-eight patients were selected, including 10 male and 18 female patients with an age range of 21–84 years (median, 54 years).

**Table 2:** **Clinical data of 28 patients with SFT**

**Case**	**Age**	**Sex**	**Size (cm)**	**Site**	**Margin status**	**Treatment**	**Local recurrence**	**MET**	**Follow-up**
1	21	F	9	Abdomen	Neg	SE	Y, multiple	Y	DOD, 52 yrs
2	74	F	9	Calf	Neg	SE	NA	NA	NA
3	59	M	13	Pelvis	Neg	SE; CT after MET	Y	Y	NED, MET 48 mo
4	55	F	9.5	Retro-peritoneum	Neg	SE; CT + RT after MET	N	Y	DOD, 13 mo
5	84	F	10	Para-tracheal	Neg	SE	NA	NA	NED, 48 mo
6	68	F	17	Lung	Neg	SE	NA	NA	NA
7	39	F	3	Thigh	Neg	SE	N	N	NED, 36 mo
8	34	M	4.1	Cheek	Neg	SE	N	N	NED, 27 mo
9	75	F	12	Neck	Neg	SE	N	N	NED, 26 mo
10	50	F	10	Uterus	Neg	SE	N	N	NED, 33 mo
11	52	F	4.7	Biceps	Neg	SE	N	N	NED, 32 mo
12	52	M	6.3	Lung	Pos	SE	N	N	NED, 17 mo
13	56	M	16	Pelvis	Neg	SE	N	N	NED, 12 mo
14	53	F	4.5	Pleura	Neg	SE	N	N	NED, 21 mo
15	71	M	NA	Lung	NA	SE	NA	NA	NA
16	39	M	2	Mastoid	Pos	EMB + SE; SE + RT after recurrence	Y	N	NED, 7 mo after treatment of recurrent disease
17	44	F	2.5	Orbit	Neg	SE	Y	N	NA
18	55	M	13	Lung	Neg	SE	N	N	NED, 9 yrs
19	63	M	4	Lung	Neg	SE	NA	NA	NA
20	45	F	7.5	Mesenterium	Neg	SE	N	N	NED, 10 yrs
21	33	F	9	Calf	Pos	SE	N	N	NED, 23 mo
22	67	F	NA	Buttock	NA	SE	N	N	NA
23	68	F	14	Pleura	NA	SE	N	N	NED, 8 mo
24	76	M	7	Abdomen	Neg	SE	NA	NA	NA
25	69	F	NA	Lung	NA	SE	NA	NA	NA
26	43	M	9.5	Back	Neg	SE	NA	NA	NA
27	42	F	1.6	Head	Neg	SE	NA	NA	NA
28	42	F	5.5	Knee	Neg	SE	N	N	NED, 9 yrs

*NA* = data not available; *SE* = surgical excision; *MET* = metastasis; *EMB* = embolization; *DOD* = died of disease; *NED* = no evidence of disease; *AWD* = alive with disease; *CT* = chemotherapy; *RT* = radiotherapy; *Y* = yes; *N* = no.

Tumors arose in the lung and pleura (9, 32%), deep soft tissue of the lower extremities (5, 18%), head and neck (5, 18%), abdomen (3, 11%) and pelvis (2, 7%). Other sites were uterus, retroperitoneum, deep soft tissue of the upper extremity and deep soft tissue of the back (1 case each).

Surgical excision was performed in 28 patients, 1 after prior embolization. Resection margins were positive in 3 patients, negative in 21 patients, and not reported in 4 cases. One patient with positive margins (case 16) and a subsequent local recurrence received surgery combined with radiotherapy for his recurrent disease and had no evidence of disease at 7 months. The other 2 patients with positive resection margins did not receive post-surgical therapy and had no evidence of disease at 17 and 23 months.

Recurrences occurred in 4 instances (14%) including 3 patients with negative margins and the above mentioned case 16 with positive margins. One of the 3 patients with negative margins (case 1) had surgical therapy for several local recurrences and metastatic disease. One patient (case 3) received surgery for local recurrence and chemotherapy after discovery of metastatic spread. Fifteen patients had no recurrences, and of 9 patients, data about local recurrences were not known.

Metastatic disease occurred in 3 patients, including case 1 and case 3 mentioned above. One patient (case 4) received combined radio-chemotherapy after discovery of metastases. Sixteen patients showed no metastatic disease and of the remaining 9 cases, data were not known.

Two patients died of metastatic disease 13 months and 52 years after the initial diagnosis (case 4 and 1, respectively). In case 1, recurrences and metastases were histologically proven.

Currently, 16 patients have no evidence of disease with a median follow up of 29.5 months (range 7 – 120 months), including 3 patients with long term disease-free follow up. Ten patients were lost to follow-up or no follow-up data could be obtained.

### Gross findings

Tumor size ranged from 1.6 – 17 cm (median, 9 cm). Grossly, the tumors had a (multi)nodular configuration with a coarse white-grayish cut-surface. Myxoid areas were apparent in some cases.

### Microscopic findings

**Figure 1:**
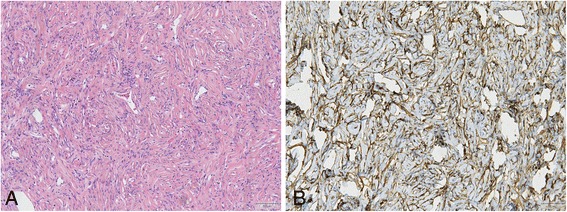
**Typical features of SFT visible in all cases (A, HE 10x) and immunohistochemical positive staining of CD34 (B, 10x).**

Histologically, most of the tumors were circumscribed and a (incomplete) pseudo capsule was sometimes detected. In few cases, focal infiltration of adjacent structures was seen. All cases showed typical features of SFT with a patternless architecture of alternating hypo- and hyper cellular areas of spindle-shaped cells (Figure 1A). A round cell component was found in 2 cases (case 17 and recurrences of case 1). The latter case showed additionally a fascicular growth pattern in the recurrences. The nuclei were relatively uniform, spindled or oval-shaped. Nuclear atypia was focally obvious in case 12 and giant cells were detected in case 8. The mitotic rate ranged from 0/10 HPF to 30/10 HPF (with no differences between patients with or without metastatic disease, Table 3). Hemangiopericytoma-like vessels were visible in all cases, partly with hyalinization of the vessel walls. A variable collagenous background was existent and case 5 possessed amianthoid fibers. Pseudocystic changes were seen in myxoid areas of 5 cases and small areas of necrosis were present in 3 cases. Mature adipocytes were a component in 1 case being an example of a morphologically malignant fat-forming SFT which was published previously [15].

**Table 3:** **Results translocation analysis, mitotic index and immunohistochemical staining**

**Case**	**Fusion**	**mit.**	**CD34**	**CD99**	**EMA**	**SMA**	**S100**	**BCL2**	**STAT6**	**EGR1**	**Others**
1	Ex4-Ex3	8/10	+	+	-	-	-	+	+	1	desmine -
2	Positive	3/10	+	+		+	+	+	+	3	desmine -, CK-pan -
3	Ex4-Ex3	3/10	+			-		+	+	1	desmine -
4	Negative	0/10	-	+	-	+	-	+	+	1	desmine -
5	Ex4-Ex3	0/10	+	+		-	-	+	+	1	desmine -, CK-pan -
6	Ex4-Ex3	4/10	+	w+			-	+	+	2	CK-pan +
7	Negative	1/10	+	+	-	-	-	+	+	5	CK-pan -
8	Positive	0/10	+	+		-		+	+	2	CK-pan -
9	Ex7-Ex3	0/10	+	+				+	+	2	CK-pan -
10	Negative	1/10	+	+	-	-	-	+	+	3	desmine -, CK-pan -
11	Negative	0/10	+	+		-	-	+	+	2	desmine -, CK-pan -
12	Ex4-Ex3	1/10	+					+	+	4	desmine -
13	Negative	0/10	+	+	f+	-	-	+	+	3	desmine -
14	Ex4-Ex3	0/10	+	+				+	+	1	
15	Ex4-Ex3	0/10	+	+		-	-	w+	+	NA	
16	Ex6-Ex17	30/10	+	+				+	+	3	desmine -
17	Ex6-Ex18	13/10	+	+	-		-	+	+	1	desmine -, CK-pan -
18	Ex4-Ex3	0/10	+			-	-	+	+	2	desmine f+, CK-pan -
19	Ex4-Ex3	0/10	+			-	-		+	1	desmine -
20	Negative	1/10	+			-	-		+	1	desmine -, CK-pan -
21	Ex6-Ex18	0/10	+					+	+	1	
22	Ex6-Ex3	0/10	+			w+	-		+	1	desmine -
23	Ex4-Ex3	8/10	+	+		-	-	+	+	1	desmine -
24	Negative	1/10	+	+				+	+	3	desmine -, CK-pan -
25	Ex4-Ex3	1/10	+						+	1	
26	NA	0/10	+						+	5	
27	Ex6-Ex18	0/10	+						+	2	
28	NA	6/10	+	-			-		+	5	desmine -, CK-pan -

*Mit*., mitotic figures/10 HPF; *w+*, weakly positive; *f+,* focally positive; *NA* = not assessable EGR1: 0 = negative; 1 = <5% nuclei +; 2 = 5-25% nuclei +; 3 = 26-50% nuclei +; 4 = 51-75% nuclei +; 5 = 76-100% nuclei +.

Fusion-positive cases are characterized by exon junctions, except for case 8 showing a complex junction indicated as positive; negative, no fusion detected.

### Immunohistochemical findings

**Figure 2:**
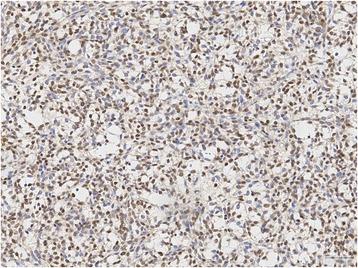
**Immunohistochemical staining of STAT6 showing nuclear positivity in all cases (20x).**

**Figure 3:**
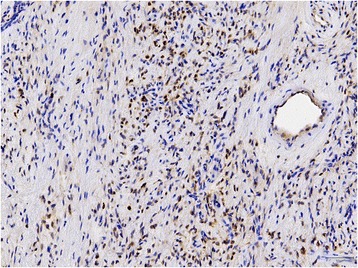
**Low level variable expression of EGR1 was seen in all samples (20x).**

Immunohistochemical staining results are summarized in Table 3 and Table 4. All 28 cases were stained for STAT6 and all of them showed diffuse and strong nuclear positivity (Figure 2; 28/28, 100%). All 28 cases were stained for EGR1 (Figure 3). Most cases showed less than 25% nuclear positivity (12/28 cases score 1, 6/28 cases score 2). Few cases had more than 25% of all nuclei stained (5/28 cases score 3, 1/28 cases score 4, 3/28 cases score 5). Scoring of one case was not possible due to limited available tissue. All dedifferentiated liposarcomas (0/5 pos), deep benign fibrous histiocytomas (0/5 pos), sarcomatoid mesotheliomas (0/5 pos), low grade fibromyxoid sarcomas (0/7 pos), schwannomas (0/5 pos), malignant peripheral nerve sheath tumors (0/4 pos), gasto-intestinal stroma cell tumors (0/5 pos), synovial sarcomas (0/5 pos) and leiomyomas (0/6 pos) showed no nuclear staining for STAT6. EGR1 showed variable expression in the different tumor samples, as depicted in Table 4.

**Table 4:** **Immunohistochemical staining results control samples**

**Soft tissue tumor**	**EGR1**	**STAT6 (pos/total (%))**
Dedifferentiated liposarcoma	1,2,3,3,3	0/5 (0%)
Low grade fibromyxoid sarcoma	1,2,2,2,2,3,3	0/7 (0%)
Deep benign fibrous histiocytoma	3,4,4,4,4	0/5 (0%)
Malignant peripheral nerve sheath tumor	2,2,3,4	0/4 (0%)
(Sarcomatoid) mesothelioma	1,2,2,3,3	0/5 (0%)
Schwannoma	2,3,3,3,4	0/5 (0%)
Gastro-intestinal stroma cell tumor	2,3,3,4,4	0/5 (0%)
Synovial sarcoma	2,3,3,3,4	0/5 (0%)
Leiomyoma	1,1,2,2,2,3	0/6 (0%)

CD34 (Figure 1B) was positive in 24 of 25 stained cases (96%) and CD99 in 17 of 18 stained cases (94%). Bcl-2 showed expression in 100% of the stained samples. EMA, SMA and S100 were negative in most of the cases (83%, 80% and 94%, respectively). One case each was (at least focally) positive for pan-keratin and desmin (Table 3).

### Molecular genetics findings

**Figure 4:**
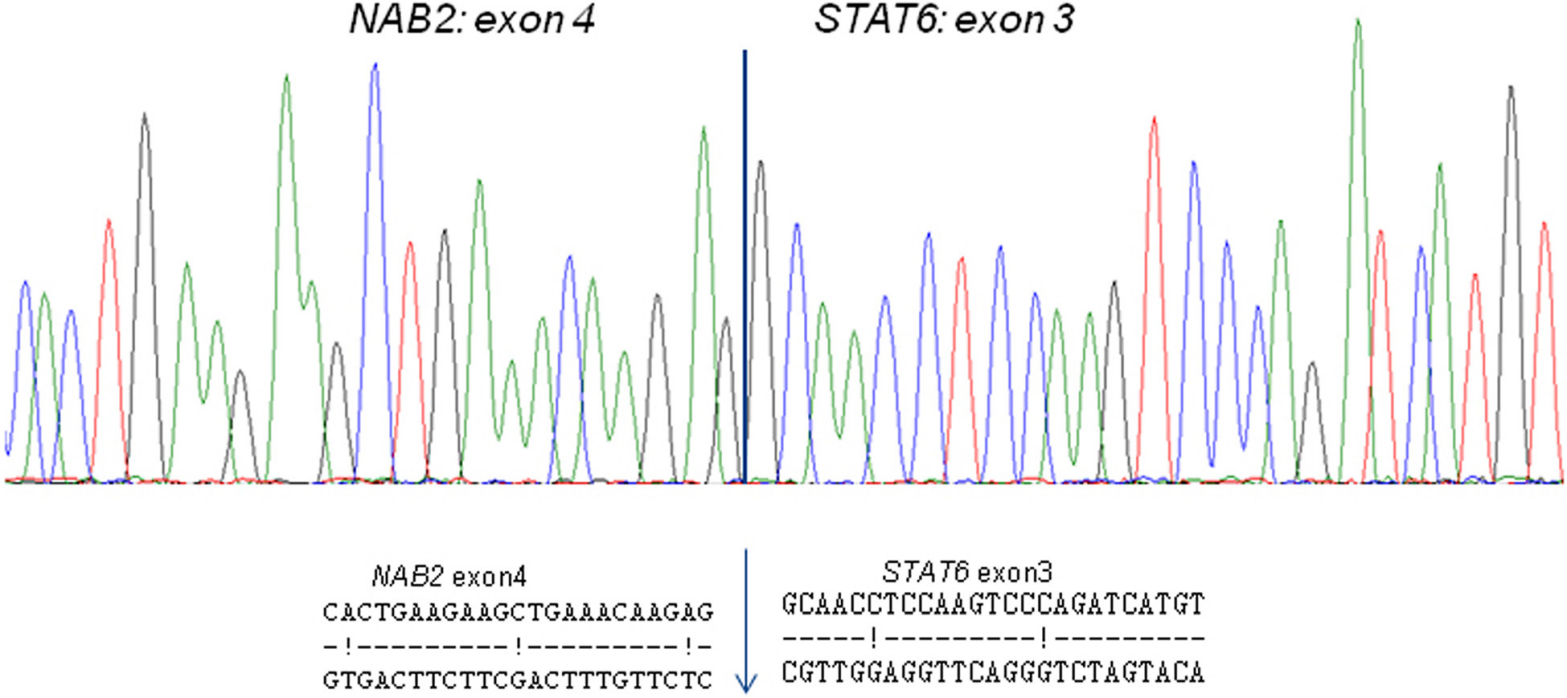
**Sequence of the RT-PCR product shows a fusion between exon 4 of NAB2 and exon 3 of STAT6 in the chimeric transcript of 12 cases.**

Results of molecular analysis are summarized in Table 3. *NAB2-STAT6* fusion transcripts were found in 19/28 cases (68%). Most fusions occurred between *NAB2* exon 4 and *STAT6* exon 3 (11/19, 58%; Figure 4). Nine of them were detected in lung and pleura lesions. Three cases (16%) had the isoform *NAB2* exon 6 with *STAT6* exon 18. Two of them were located in the head and neck region. Single cases showed fusion variants of *NAB2* exon 6 and *STAT6* exon 17, *NAB2* exon 7 and *STAT6* exon 3 and *NAB2* exon 6 and *STAT6* exon 3. Two tumors (case 2 and case 8) harbored a fusion, but due to complex breakpoints with a possible inversion the exact exons could not be verified. In 7 tumors, no *NAB2-STAT6* fusion was found (7/28, 25%). In case 26 and case 28, adequate interpretation of RT-PCR results was not possible due to the presence of complex breakpoints.

## Discussion

In 1942 and 1949, Stout and Murray defined hemangiopericytoma as a neoplasm with prominent branching vessels surrounded by pericytic tumor cells [16,17]. The lesions included in their studies comprised true pericytic tumors, nowadays termed myofibroma/myopericytoma, and conventional hemangiopericytoma, which was afterwards accepted as cellular variant of SFT [18]. The unified entity SFT now also contains the lipomatous variant, giant cell angiofibroma, and at the malignant end, dedifferentiated SFT showing abrupt transition from classical areas into high-grade sarcoma [1].

With the advent of whole-genome sequencing, the fusion gene *NAB2-STAT6* has been detected as the driver mutation of SFT [2,9,10,14]. *NAB2* and *STAT6,* adjacent genes on 12q13, fuse following genomic inversion of *STAT6* with consecutive transcription in a common direction [2,9]. Expression of the chimeric protein NAB2-STAT6 leads to activation of EGR-mediated signaling via distorted NAB2 activity. Modulation of STAT6 dependent gene expression seems to be an alternative mechanism but was shown to play a minor role [2,9].

*NAB2-STAT6* fusion is a distinct molecular feature of SFT since it has not been detected in other tumors (so far) and its frequency ranges from 55% to 100%, irrespective of dignity [2,9,13,14]. There are several fusion variants, with conjunction of *NAB2* exon 4 - *STAT6* exon 3 and *NAB2* exon 6- *STAT6* exon 17 being the most common [2,13,14]. In our study, 68% of SFTs carried a *NAB2-STAT6* fusion transcript, mostly with a fusion between *NAB2* exon 4 and *STAT6* exon 3 in accordance with the results by Mohajeri et al. and Barthelmess et al. [13,14]. Negativity for this fusion in 25% of our cases could possibly be explained by alternative or complex genetic rearrangements involving other exons or inversions and deletions, not detectable with our primer combination. In 2 cases (7%), adequate interpretation of RT-PCR results was not possible due to the presence of complex breakpoints. Furthermore, in single cases other fusion genes have been reported, e.g. *STAT6-TRAPPC5* [14] and probably additional fusion genes will be detected in the future.

Doyle et al. demonstrated nuclear expression of STAT6 in 98% of a large series of SFTs indicating the presence of the NAB2-STAT6 fusion protein in the nucleus [11]. STAT6 is therefore a highly sensitive and specific immunohistochemical marker for SFT [10-13,19,20]. This is in concert with our results, where 100% of the cases showed strong and diffuse nuclear positivity for STAT6 in comparison to the control group being 100% negative. Thereby, STAT6 was diffusely expressed in 7 tumors without a detected *NAB2-STAT6* fusion, suspecting limitations in our RT-PCR approach in which *NAB2-STAT6* fusions could be missed as mentioned above. Potential diagnostic pitfalls could be STAT6 expression in, for example, the morphologic mimics deep benign fibrous histiocytoma and dedifferentiated liposarcoma, especially in retroperitoneal and abdominal localization [11]. As known, MDM2 and CDK4 immunohistochemistry or *MDM2* FISH are useful in identifying dedifferentiated liposarcoma. Expression of STAT6 in ca. 10% of dedifferentiated liposarcomas is based on amplification of the corresponding gene located in proximity to *MDM2* and *CDK4* [11,12,21,22]. Deep benign fibrous histiocytoma may also show a hemangiopericytoma-like vasculature and may express CD34 [1]. In such cases, molecular analysis of *NAB2-STAT6* may be helpful. Other differential diagnoses and their histopathological characteristics are listed in Table 5.

**Table 5:** **Differential diagnoses of SFT (in accordance with Fletcher et al. and Doyle et al.** [1,11]**)**

	**Histopathological characteristics**	**IHC**	**Genetic alterations**
Dedifferentiated liposarcoma	lipogenic component with atypia	MDM2 +, CDK4 +	amplification *MDM2*
	atypia in dediff. component can be mild	STAT6 −/+	
Deep benign fibrous histiocytoma	storiform or short fascicles, branching vasculature	CD34 +/−, SMA f+/−, STAT6 −/+	
Spindle cell lipoma	short stubby nuclei, ropey collagen	CD34+	*RB1*deletion
	variable proportions of fat		
Cellular angiofibroma	short stubby nuclei, wispy collagen	CD34 +/−, desmin −/+	*RB1* deletion
	numerous (hyalinized) vessels	SMA −/+,	
Mammary type myofibroblastoma	(long) fascicles, short stubby nuclei	CD34 +, desmin +	*RB1* deletion
	broad bands collagen	SMA −/+,	
Myofibroma	biphasic, immature spindle cells and mature myoid cells, bluish matrix, branching vessels	SMA +/−, desmin −/+, CD34 −/+	
Dermatofibrosarcoma protuberans	storiform, short fascicles, uniform spindle cells, “honeycomb” appearance of fat	CD34 +	*COL1A1-PDGFB*
Monophasic synovial sarcoma	cellular fascicles, uniform spindle cells	TLE1 +, EMA +, CK +	*SYT-SSX1/2*
Cellular schwannoma	short bundles, interlacing fascicles	S100 +	*NF2* mutations
	tapered nuclei, hyalinized (ectatic) vessels		
Perineurioma	storiform, whorled growth, bipolar cells, uniform oval or tapering nuclei	CD34 +/−, EMA +, Claudin-1−/+, GLUT1 −/+	
Low-grade fibromyxoid sarcoma	alternating hypo- and more cellular fascicles and whorls, fibrous and myxoid background, bland spindle cells	MUC4 +, EMA +	*FUS-CREB3L1/2*
Inflammatory myofibroblastic tumor	fascicles of myofibroblasts, myxoid or	SMA +/−, desmin +/−	*ALK* rearrangement +/−
	collagenous background, inflammation	ALK +/−	
Sarcomatoid mesothelioma	fascicles or haphazard distribution, atypical spindle cells	CK, EMA, D2-40, caretinin, WT1	*BAP1* mutation [23]
Inflammatory fibroid polyp	onion-skin pattern, short fascicles	CD34 +/−	*PDGFRA* mutation
of the GI tract [24] cells,	prominent vasculature, inflammatory especially eosinophils		
Soft tissue angiofibroma [25]	variably collagenous and myxoid areas	EMA +/−, CD34 −/+	*GTF2I-NCOA2* [26]
	numerous small branching capillaries		*AHRR-NCOA2* [27]

Previously, CD34, CD99 and bcl-2 were the most useful positive immunohistochemical markers for SFT. However, they are less specific and absence of CD34 does not rule out SFT [1,28-31].

As expected, CD34, CD99 and bcl-2 were positive in most of our cases. Case 4 with negative staining for CD34 and absence of a detectable gene fusion showed strong nuclear expression of STAT6 and no *MDM2* amplification, so the possibility of being a dedifferentiated liposarcoma was ruled out.

Cell line experiments supported that in contrast to the known transcriptional repressor activity of NAB2, the NAB2-STAT6 fusion protein leads to activation of EGR1 and expression of its target genes. In addition, the increased proliferation of the NAB-STAT6-expressing cell lines could be inhibited by small interfering RNA (siRNA) knockdown of *EGR1* expression [2]. This prompted us to investigate EGR1 protein expression in our tumor samples which showed low-level variable nuclear reactivity in all stained samples, including 47 control samples of possible mimickers of SFT. This result is in line with EGR1 protein activation without high-level expression mainly due to altered NAB2 function resulting in deregulation of target genes as mentioned above [2,14].

Correlation of clinicopathologic parameters and outcome is difficult. This could be due to, at least in part, relatively small cohorts of SFT studied. Therefore, no statistical correlation could be made between pathological findings and clinical data. To date, no definitive markers have been identified that classify malignant SFT. Recently, efforts have been made to define a risk assessment model based on patient age, tumor size and mitotic index with promising results [7,8]. From the genetic point of view, Mohajeri et al. [14] did not find any clinical associations including genetic changes beyond the fusion-genes. In contrast, Barthelmess et al. showed that the most common fusion variant *NAB2* exon 4-*STAT6* exon 3 corresponded to classic pleuropulmonary SFT as we found in our study: all 9 pleuropulmonary SFTs had a fusion between *NAB2* exon 4 and *STAT6* exon 3.

*NAB2* exon 6-*STAT6* exon16/17 fusions were detected in cellular soft tissue SFTs with more aggressive behavior in younger patients [13].

Three of the 18 patients (17%) of whom we had adequate clinical follow-up data (cases 1, 3, 4) behaved in a malignant fashion with metastases. Only one of them showed a high mitotic index (case 1). Locations were abdomen, pelvis and retroperitoneum in accordance with known parameters for malignant potential but sizes were below 10 cm [6-8].

Therapeutic options for more advanced or aggressive tumors are scarce and no standard modality for metastasized SFT has currently been accepted. Few chemotherapeutical drugs have been studied with different outcome. Anti-angiogenic targeted treatment shows interesting results [4,5]. The potential role of EGR1 and its downstream targets in treatment of (metastasized SFT) is not clearly defined so far [2,9].

## Conclusions

In summary, STAT6 immunohistochemistry is a powerful tool in diagnosing SFTs. Also, the identification of the *NAB2-STAT6* fusion gene can provide important diagnostic information, even in formalin-fixed and paraffin-embedded tissue or when biopsy material is limited. In accordance with Barthelmess et al., the most common fusion variant *NAB2* exon 4-*STAT6* exon 3 corresponded mostly to pleuropulmonary SFT.

EGR1 immunohistochemistry indicates low-level protein expression in accordance with EGR1 activation due to distorted NAB2 activity resulting in deregulation of EGR1 target genes.
